# Autumn larval cold tolerance does not predict the northern range limit of a widespread butterfly species

**DOI:** 10.1002/ece3.7663

**Published:** 2021-05-22

**Authors:** Philippe Tremblay, Heath A. MacMillan, Heather M. Kharouba

**Affiliations:** ^1^ Department of Biology University of Ottawa Ottawa ON Canada; ^2^ Department of Biology Carleton University Ottawa ON Canada

**Keywords:** cold distribution limits, global warming, insect, Maxent, mechanistic species distribution model

## Abstract

Climate change is driving range shifts, and a lack of cold tolerance is hypothesized to constrain insect range expansion at poleward latitudes. However, few, if any, studies have tested this hypothesis during autumn when organisms are subjected to sporadic low‐temperature exposure but may not have become cold‐tolerant yet. In this study, we integrated organismal thermal tolerance measures into species distribution models for larvae of the Giant Swallowtail butterfly, *Papilio cresphontes* (Lepidoptera: Papilionidae), living at the northern edge of its actively expanding range. Cold hardiness of field‐collected larvae was determined using three common metrics of cold‐induced physiological thresholds: the supercooling point, critical thermal minimum, and survival following cold exposure. *P*. *cresphontes* larvae were determined to be tolerant of chilling but generally die at temperatures below their SCP, suggesting they are chill‐tolerant or modestly freeze‐avoidant. Using this information, we examined the importance of low temperatures at a broad scale, by comparing species distribution models of *P*. *cresphontes* based only on environmental data derived from other sources to models that also included the cold tolerance parameters generated experimentally. Our modeling revealed that growing degree‐days and precipitation best predicted the distribution of *P*. *cresphontes*, while the cold tolerance variables did not explain much variation in habitat suitability. As such, the modeling results were consistent with our experimental results: Low temperatures in autumn are unlikely to limit the distribution of *P*. *cresphontes*. Understanding the factors that limit species distributions is key to predicting how climate change will drive species range shifts.

## Graphical abstract

We integrate organismal thermal tolerance measures into broadscale species distribution models to test the critically important but untested hypothesis that low temperatures during autumn limit insect distribution. We do this using a widespread butterfly species, *Papilio* *cresphontes*, that is actively expanding its range northward. Results across approaches were consistent in rejecting the hypothesis that low temperatures during autumn limit the distribution of *P. cresphontes*. Instead, our modeling revealed that growing degree‐days and precipitation best predict the distribution of *P. cresphontes*.

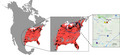

## 1 INTRODUCTION

Over the past few decades, range shifts due to climate and land‐use changes have been reported in both plants and animals. Many are experiencing a poleward and/or upward shift in distribution, pushing their northern and upper range limit to higher latitudes and altitudes (Chen et al., 2011; VanDerWal et al., 2013; but see Kerr et al., 2015). However, there has been substantial variation in the degree and direction of range shift across taxonomic groups (Chen et al., 2011; Devictor et al., 2012; Lenoir & Svenning, 2015). This variation remains difficult to explain and necessitates a better understanding of the factors influencing species’ distributions (Buckley & Kingsolver, 2012; Devictor et al., 2012; Sgrò et al., 2016; Sunday et al., 2012).

A climate change‐driven range shift in insects is often attributed to the relaxation of a harsher poleward climate since temperature acts as a physiological limitation and as a phenological cue (Paradis et al., 2008; Logan & Bentz, 1999; Root et al., 2003). For those species with northern range edges associated with temperature clines, climatic warming has frequently resulted in the colonization of suitable areas in more northern latitudes (Parmesan, 1996; Parmesan & Yohe, 2003). For example, the northern range expansion of the deer fly (*Lipoptena cervi*) in Finland is due to warmer temperatures during the summer (Härkönen et al., 2010). However, for many other species, it remains unclear how climate change has led to range shifts (Chen et al., 2011; Sunday et al., 2012).

Low temperatures throughout the year can constrain the ability of insects to persist at northern range limits as they can influence any life cycle stage (Ungerer et al., 1999). Overwinter survival has been shown to be a key factor limiting the ranges of some insects (e.g., Southern pine beetle *Dendroctonus* *frontalis,* Crozier, 2003). Overwintering insects can adopt one of multiple physiological strategies for surviving winter (Williams et al., 2015); some insects can survive internal ice formation and are termed freeze‐tolerant, some physiologically suppress the temperature at which their body fluids spontaneously freeze (the supercooling point; SCP) and are termed freeze avoiding, while others remain susceptible to chilling (termed chill susceptible) and seek relatively warm microhabitats to overwinter (Denlinger & Lee, 2010; Overgaard & MacMillan, 2017; Sinclair, 1999).

While winter represents a considerable challenge (Robinet & Roques, 2010; Williams et al., 2015), insects are also likely to be particularly vulnerable to low temperatures during autumn. Autumn is when most insects are acquiring cold tolerance through physiological adjustments (acclimatization) and have therefore not reached their peak cold hardiness (i.e., the capacity to tolerate intensity and duration of cold exposure; Tauber et al., 1986). There is also a much higher frequency of cold temperature events in autumn than in the summer (Danks, 1978). Yet, no study, to our knowledge, has considered low temperatures during autumn as a limiting factor on the geographic distributions of insects.

Regardless of season, cold exposure can cause insects to cross physiological thresholds leading to sublethal effects. At a species‐specific low temperature, most insects lose the ability for coordinated movement (a critical thermal minimum; CT_min_) after which they enter a state of complete neuromuscular silence termed chill coma (Gibert & Huey, 2001; MacMillan & Sinclair, 2011; Oyen & Dillon, 2018). Many insects can recover from this state with no immediate evidence of injury, but prolonged or severe cold exposure can cause behavioral defects, or slow or halt development (Asahina, 1970; Overgaard & MacMillan, 2017; Rojas & Leopold, 1996). Chill‐susceptible insects suffer from a loss of homeostasis at low temperatures well above their SCP, while those more tolerant to chilling survive such exposures (Overgaard & MacMillan, 2017). While freeze‐tolerant insects can survive freezing of the extracellular fluid at their SCP, they can suffer from ice‐related injuries below this temperature. By contrast, freeze‐avoidant insects cannot survive at temperatures below their SCP. Thus, the relationship between the SCP and injury/mortality is critical to determining the cold tolerance of these insects (Sinclair et al., 2015).

Cold tolerance traits, such as the CT_min_, SCP, or survival following a cold stress, frequently correlate strongly with insect distribution (Andersen et al., 2015; Bozinovic et al., 2011; Gouveia et al., 2014). For example, the CT_min_ and CT_max_ of *Drosophila* species have been used to accurately predict their current distributions (Overgaard et al., 2014). However, given the limited number of taxa in which these relationships have been explored, we have little to no ability to generalize predictions about how climate influences species’ distributions via low temperatures to other taxa (Ouimette, 2018).

Species distribution modeling is a commonly used approach to evaluate recent range shifts and forecast future shifts due to climate change. Using correlations between georeferenced occurrence records and a set of environmental variables with geospatial data, these models predict a species’ suitable habitat (Elith et al., 2011). While the approach is useful for many applications, these models often violate key assumptions, such as predicting habitat suitability in novel conditions and omitting key biotic variables known to influence geographic distributions such as species interactions (Briscoe et al., 2019; Tingley et al., 2014).

One way that has been demonstrated to improve the accuracy of these models is to incorporate physiological variables or stress tolerance thresholds (e.g., metabolic needs, thermal limits; Kearney & Porter, 2009; Overgaard et al., 2014). By using variables derived directly from these physiological traits, the underlying processes explaining the species’ distribution are thought to be better incorporated into these “mechanistic” or “process‐based” models (Kearney & Porter, 2009). In some contexts, these models have been shown to be more accurate than (Peterson, 2011), or complement weaknesses of (Martínez et al., 2015), correlative models when modeling the fundamental niche and can strengthen predictions about future distributions (Buckley et al., 2011; Evans et al., 2016; Kotta et al., 2019; Martínez et al., 2015). However, these experimentally derived variables can be time‐consuming to develop and thus not feasible to do for large assemblages of species or over broad ranges (Evans et al., 2016; Peterson, 2011). It can also be difficult to find traits that are predictive and functional in the species’ environment (Kearney & Porter, 2009).

Here, we test the hypothesis that low temperatures during autumn are the limiting factor of the northern range edge of the widespread butterfly, the Giant Swallowtail, *Papilio cresphontes* (Cramer, 1777). Specifically, our two main objectives are to (a) find relevant cold tolerance thresholds of *P*. *cresphontes* collected in late summer; and (b) determine the relative importance of these thresholds on the geographic distribution of *P*. *cresphontes*. Simultaneously, we aim to determine the cold tolerance strategy of larvae at the northern range limit. Determining the species’ cold tolerance strategy improves our knowledge about the importance of low temperatures on *P*. *cresphontes* survival and helps identify which low‐temperature thresholds are likely to be most relevant at the species’ northern range. In this study, we focus on the pre‐overwinter life stage (i.e., larval stage), which is in contrast to most other studies that have determined the cold tolerance strategy of the life stage that overwinters (Radchuk et al., 2013). To determine the importance of low temperatures at a broad scale, we model the geographic distribution of *P*. *cresphontes* with the cold tolerance parameters we generated experimentally, as well as other factors hypothesized to limit the distribution of butterflies (Table S1).

*Papilio cresphontes* has undergone a rapid expansion over the past decade and now occurs as far north as Ottawa, Ontario, Canada. Finkbeiner et al. (2011) hypothesized that the range expansion into New York state from 2000 to 2010 was due to the disappearance of frost (as defined meteorologically) in September. Based on an initial assessment from a few field observations, which were not drawn from commonly used thermal limits, they showed individuals could survive a single exposure to temperatures just below freezing (0°C). However, to effectively reject the hypothesis, a more in‐depth study of the impact of low temperature on *P*. *cresphontes* using measurements of thermal limits is warranted.

To establish the larval cold tolerance strategy, we measured three common cold‐induced physiological thresholds: SCP, CT_min_, and survival following cold exposure. To gain a better estimate of the cold tolerance strategy, thresholds (SCP and low‐temperature survival) were measured for two generations and multiple sites across a latitudinal gradient at the northern range limit. Latitude has the potential to affect cold tolerance due to its correlation with climate (e.g., photoperiod, temperature; Sømme, 1982; Tanaka, 1996, but see Yoshio & Ishii, 2001). Therefore, latitude could affect when and to what extent organisms are cold hardy. Combined, these experiments were used to identify a potentially relevant low‐temperature threshold of larvae collected at the northern range edge. We examined the importance of low temperatures at a broad scale by comparing species distribution models of *P*. *cresphontes* based only on environmental data derived from other sources to models that also included the cold tolerance parameters generated experimentally.

## 2 MATERIALS AND METHODS

### 2.1 Study system

**FIGURE 1:**
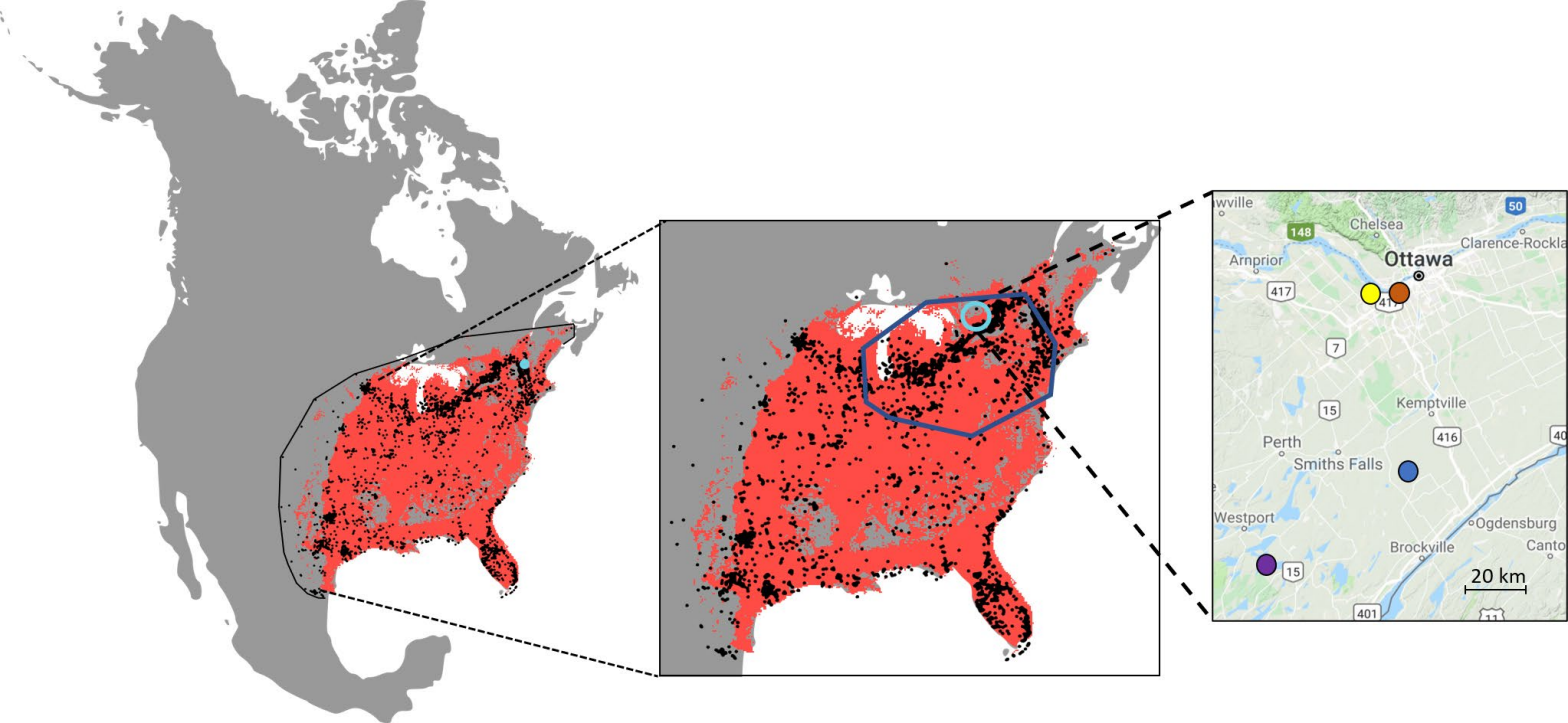
The geographic distribution and northern range of *Papilio cresphontes*. The first panel shows the entire range of *P*. *cresphontes* encompassed by the minimum convex polygon (thin black line, i.e., the background extent used in the modeling). The second panel shows the northern range defined in this study, denoted by the solid blue line. The blue circle indicates the location of the study region. In the first two panels, the occurrence points (black) are shown against the predicted habitat suitability (red) based on the full range mechanistic model. The threshold for suitability was based on the threshold selection metric in Maxent that balances rates of omission error in the training data, fractional predicted area (i.e., proportion of cells predicted to be suitable), and the cumulative threshold value. The third panel shows the field sites where larvae were collected at the northern range limit: The blue dot indicates the Brockville site (44.84952, −75.75226), purple indicates the Queen's University Biological Station site (44.56747, −76.32454), brown indicates the Mud Lake site (5.37192, −75.79451), and yellow indicates the Shirley's Bay site (45.36546, −75.88302)

The Giant Swallowtail (*Papilio cresphontes*) butterfly is a member of the Papilionidae family. The species’ range extends from Costa Rica throughout the North American continent. The bulk of the population is concentrated in the Eastern United States. It ranges as far north as the Ottawa region, Ontario, Canada (Figure 1).

*Papilio cresphontes* larvae undergo five instars before entering the chrysalis stage (Bullock & Pelosi, 1991). In Ontario, there are two generations with flights occurring from May to July and again from late July to late September (Layberry et al., 1998). The larval stage lasts for 3–4 weeks and pupae formed in the summer emerge after 10–12 days, but those that develop in autumn will remain as pupae until spring. The overwintering pupae undergo winter diapause, a state of lowered metabolism, with reduced respiration, and no feeding or growth occurs (Scott, 1997). Once eclosed, adults live for 6–14 days (Layberry et al., 1998).

*Papilio cresphontes* is known to use plants of the Rutaceae family as their primary food source in the larval stage. In Ontario, those plants consist mainly of Northern Prickly Ash (*Zanthoxylum americanum*) and hop tree (*Ptelea trifoliata*). The adults are generalists and will gather nectar from most flowering plants, for example, goldenrod (*Solidago*), swamp milkweed (*Asclepias incarnata*), and azalea (*Rhododendron* *azaleastrum*) (McAuslane, 2009).

### 2.2 Cold tolerance experiments

#### 2.2.1 Experimental overview

**FIGURE 2:**
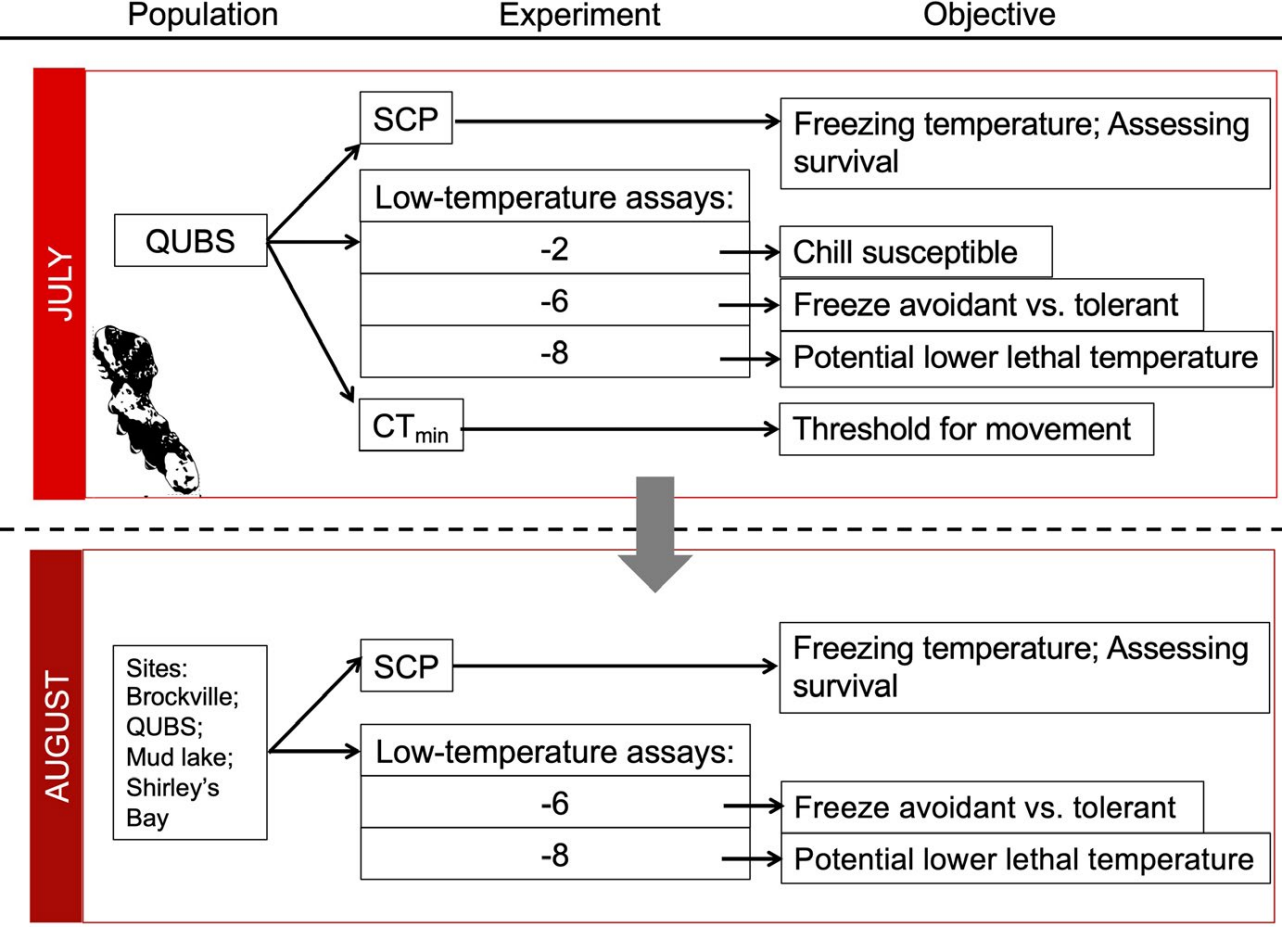
Overview of cold tolerance experiments conducted in this study. Shown are the supercooling point (SCP), low‐temperature survival assays, and critical thermal minimum experiments (CT_min_). Sites included in the experiments are as follows: Brockville, Queen's University Biological Station (QUBS), Mud Lake, and Shirley's Bay. For number of larvae in each experiment, refer to Table S1

To determine the impact of low temperatures on *P*. *cresphontes* larval survival and developmental success, three experiments were conducted (Figure 2; Figure S1). The first experiment was done to determine the cold tolerance strategy of the larvae by measuring the SCP in relation to cold survival. Additionally, since the results from this initial experiment were not enough to clearly define the cold tolerance strategy, further low‐temperature survival assays (i.e., the ambient exposure of larvae to low temperatures for an extended period of time) were conducted to better determine the impacts of prolonged low‐temperature exposure on survival and developmental success. A third experiment was conducted to measure the CT_min_.

The SCP and low‐temperature survival assays were measured for both generations and multiple sites (*n* = 4) across a latitudinal gradient at the northern range limit (Figures 1 and 2).

#### 2.2.2 Field sampling

To gain a better understanding of the cold tolerance strategy, larval sampling occurred during the two *P*. *cresphontes* generations: July and August 2018. A total of 117 larvae were collected from four sites around Ottawa, Ontario, Canada (Mud Lake, Shirley's Bay, Brockville, and Queens University Biological Station (QUBS)) spanning a 100 km latitudinal gradient at the northern range limit of *P*. *cresphontes* (Figure 1). These four sites were accessible and had a higher abundance of larvae than other sites. However, we note that this species is difficult to find because it is cryptic and at low density in this region, especially in its second generation. As such, our sample sizes in these experiments are lower than other similar physiological experiments.

Until the start of the experiments, captured individuals in July were provided with a fresh supply of *Z*. *americanum* and kept in an LTCP‐19 Biochamber at the University of Ottawa, Canada (45.4233, −75.6832), on a 21°C/25°C 15‐hr: 9‐hr L:D cycle with light‐intensity peaking at noon. These conditions were chosen to match the average environmental conditions in July for the Ottawa region. To replicate the conditions required for larvae to prepare to overwinter, the chamber parameters were modified weekly for the August generation to match the conditions from August to October (meteomedia.ca; Figure S1). Over the time spent in the chamber, peak daily temperatures reached a maximum of 25°C and a minimum of 15°C overnight at the beginning, and by the end, daytime temperatures only reached 10°C and a minimum of 6°C overnight (Figure S1). Likewise, day length fell from 14.5 hr to 11 hr. To validate our treatments, we recorded the realized climate regime in the environmental chamber using a temperature logger (HOBO Pendant; Onset Computer Corporation, Bourne, MA). We note here that there was no evidence of parasitism in any of our experiments.

#### 2.2.3 Experimental details

##### Experiment 1: Supercooling point (SCP) test

To identify the cold tolerance strategy of *P*. *cresphontes* larvae, we quantified the SCP and survival following a freezing event in the first generation (i.e., the July generation; *n* = 27 from QUBS; Figure 2; Figure S1). The SCP test was done on larvae in their final instar (median mass = 608.5 g (372.7 *SD*)) and following the recommended methodology of Sinclair et al. (2015). Before being cooled, the larvae were weighed with an ultra‐micro‐Sartorius scale and placed in individual 15‐ml centrifuge tubes. Body temperatures were measured using type K thermocouples in direct contact with the larvae and linked to a Pico Tech‐TC‐08 data logger. The vials were then suspended in a refrigerated circulator (21 L with advanced controller, VWR) filled with a mixture of ethylene glycol and water, and cooled down at 0.1°C per minute from 21°C. The test lasted between 200 and 300 min until half the specimens had reached SCP. After the SCP test, larvae were then moved to individual containers inside the LTCP‐19 Biochamber at temperature and photoperiod described above (section ii). Larvae were monitored daily until death or emergence. Individuals were considered dead if they did not react to physical stimuli: Larvae were poked with a stick, sprayed with water, and then shaken.

To get a more accurate estimate of SCP, another SCP test was done on the second generation using larvae in their final instar (i.e., the August generation: *n* = 29, median mass: 387.3 g (287.1 *SD*), QUBS population, Figure 2, Table S2; Figure S1). The individuals from the August generation were subjected to the same cooling protocol as described above but were kept submerged until all of the larvae had frozen. This was done to obtain the full distribution of SCP values. We note here that larval mass was not significantly different between the two generations (*n* = 41 (QUBS); Mann–Whitney = 122, *p* = .07).

##### Experiment 2: Low‐temperature survival assays

To identify the consequences of chronic low temperature likely to be experienced by the larvae before pupation, three low‐temperature survival assays were conducted (Figure 2; Figure S1). The duration and temperature of the assays were chosen based on the SCP determined in the first experiment and on meteorological data in the Ottawa area representing the developmental period before pupation. In the Ottawa region, this period corresponds to the month of October. We considered temperatures from 2012 to 2017, which correspond with the timing of the recent range expansion of *P*. *cresphontes* into the Ottawa area (Ontario Butterfly Atlas: www.ontarioinsects.org/atlas_online.htm) and used the “OTTAWA CDA RCS” weather station (45.38, −75.71). For this time frame, the mean and lowest temperatures recorded during frost events (as determined meteorologically: the span of time in which the temperature is below 0°C for more than one hour) were −1.33°C and −6.3°C, respectively, and the average frost time was 6.24 hr. Consequently, and in conjunction with the measured SCP of −5.6°C determined in the first experiment, trials were run at −2°C, −6°C, and −8°C for 7 hr each.

To determine whether larvae were chill susceptible, the first assay was done at −2°C (Figure 2). Mortality at this temperature (i.e., above the SCP; see results) would indicate this species is chill susceptible, whereas survival would confirm the ability of larvae to tolerate exposure to low temperatures above their SCP, thus making them chill‐tolerant or freeze‐avoidant. The second test was done at −6°C to distinguish between freeze‐avoidant and freeze‐tolerant strategies (Figure 2). Considerable mortality near the mean SCP would mean larvae are freeze‐avoidant, whereas high survival would mean they are more likely freeze‐tolerant. Finally, to find a potentially ecologically relevant low‐temperature threshold causing high mortality (i.e., a potential lower lethal limit), we also tested larval survival at the lowest recorded temperature for October at this weather station from 2012 to 2017 (i.e., −8°C). Exposure at this temperature tests the maximum larval cold tolerance and their resistance to freezing since it is well below the mean SCP obtained in the previous experiment (i.e., −5.77°C; Figure 2).

The low‐temperature assays were done in an environmental growth chamber (Biochambers Model LTCB‐19) for both larval generations and across multiple sites (Figure 2; Table S2). The −2°C test was not repeated in August because (a) there was high survival with the July individuals (see Section 3); (b) a low number of specimens were collected in the field; and (c) this temperature was unlikely to be a limiting factor for larval survival and thus was not a priority to test. Larvae were weighed and measured before being placed in individual containers inside the chamber. The chamber temperature was brought down from 21°C to the predetermined test temperature at a rate of 0.1°C per minute. Once the desired minimum temperature was reached, the temperature remained constant for seven hours before being increased back to 21°C at the same rate. Larvae were then left to recuperate. They were allowed to feed on freshly harvested *Z*. *americanum* leaves during the trial and were checked daily for mortality. Death was determined when larvae did not react to physical stimuli: Larvae were poked with a stick, sprayed with water, and then shaken. All larvae that survived to pupation were kept until emergence. Surviving pupae from the second generation were moved outside to overwinter in a mesh enclosure.

##### Experiment 3: Critical thermal minimum (CT_min_)

To identify the temperature at which larvae lose coordination, a CT_min_ test was done on twenty larvae that were caught in July from the QUBS site, following the method of Andersen et al. (2015). The CT_min_ test was only done on July individuals since it required a higher number of specimens than the other tests and our sample size was limited.

Once caught, we weighed and measured larvae before being placed in 50‐ml Eppendorf vials. These vials were then mounted on an aluminum rack, which was submerged in a refrigerated circulator (21 L with advanced controller, VWR) containing a mixture ethylene glycol and water. The bath was cooled from 21°C to −20°C at a rate of 0.2°C per minute. The tubes were periodically (i.e., approximately every 2 min) prodded using a plastic spoon during the cooling process to stimulate movement. The CT_min_ was determined when individuals fell on their sides and were no longer able to stand. Larvae were kept in the bath until the final larva fell, at which point the whole rack was removed from the solution and allowed to warm back up. Larvae were then left to recuperate in a growth chamber (Biochambers Model LTCB‐19) at the average conditions in July, as described above. Larvae were checked daily for mortality.

#### 2.2.4 Statistical analysis

To compare survival rates of larvae of the July generation that froze to those that did not freeze during the SCP experiment, a chi‐square test was used. As body mass was not related to larval survival in the July generation (*n* = 13, *z* =− 0.85, *p* = .39; binomial generalized linear model), we did not consider it further in the analysis. The rate above and below the SCP for the August generation could not be compared since all larvae were exposed to low temperatures until SCP was reached (i.e., no survival rate was calculated above SCP). As body size can influence water content, and thus affect SCP, we also report correlations between body mass and SCP for each generation.

To test whether mean SCP differed across sites for the August generation, an ANOVA was run. Since there was no significant difference in mean SCP across sites (*df* = 3, *F*‐value = 0.94, *p* = .44), site‐level data were pooled. The assumptions of normality and homogeneity were evaluated using the Shapiro–Wilk test and the Brown–Forsythe for homogeneity of variance test, respectively.

To compare the larval survival rate and the rates of pupation and adult emergence between generations and sites for the low‐temperature assays, a chi‐square test was used. When assumptions for the chi‐square test were not met, we used Fisher's exact test. There was no impact of site on survival rate or developmental success in any assay (Table S3), so site‐level data were pooled. All analyses were conducted in R (version 3.4.1).

### 2.3 Distribution modeling

#### 2.3.1 Overall approach

To determine the limiting factors of the current geographic distribution of *P*. *cresphontes*, we modeled its range at two different spatial extents: northern range and the entire distribution (Figure 1), and used two different modeling approaches. We wanted to determine whether the relative importance of variables differs between the northern range and the entire distribution. We defined the northern range as the area encompassing the upper 50th percentile of occurrences based on both latitude and longitude in the northeastern part of the range (Figure 1). The 50th percentile represented a clear threshold in the latitudinal distribution of occurrences. To assess whether the physiological‐based cold tolerance metrics were significant predictors of *P*. *cresphontes* distribution, the modeling was done using two approaches: correlative and mechanistic. The correlative approach was based on environmental data derived from other sources (e.g., remote sensing, weather stations), whereas the mechanistic approach additionally included parameters derived from the cold tolerance experiments.

For both approaches, we tested the importance of the cold tolerance strategy parameters by comparing (a) model fit with and without them and (b) the proportion of suitability explained among a number of climate‐related factors hypothesized to constrain the range of *P*. *cresphontes* at the landscape scale.

#### 2.3.2 Data

Models were built using all available occurrence records for North America from e‐butterfly (www.e‐butterfly.org, accessed on July 2018, 2,255 records), the Global Biodiversity Information Facility (GBIF; https://www.gbif.org, accessed on September 2018, 3,420 records), and Moths and Butterflies of North America (www.butterfliesandmoth.org, accessed on October 2018, 1,206 records) from 1980 to 2018. This time frame was chosen to match the time frame of the available environmental variables. Records that were obviously inaccurate (e.g., in the middle of the ocean) or had no geographical coordinates were removed. To reduce the likelihood of an occurrence being based on the same individual being observed on multiple occasions, duplicate records across data sources were also removed. To reduce sampling bias, we used a low‐end estimate of dispersal ability (1 km) and randomly selected a single occurrence across data sources per 1 km^2^. In total, 3,510 and 1,457 occurrences were used for the North American and the northern range extent, respectively. Occurrences were then mapped onto a North America Lambert Conical Conic projection.

Sixteen environmental variables were initially considered for the model building based on previous work done on butterflies (Table S1; Araújo & Luoto, 2007; Finkbeiner et al., 2011; Roland & Matter, 2016). The variables included represented both the direct and indirect effects (i.e., via a species’ interaction) climate can have on a species’ range (Table S1). Variables included low‐ and high‐temperature requirements (i.e., mean temperature of the coldest month and extreme maximum temperature), precipitation, measures of heat accumulation (i.e., growing degree‐days), vegetation (i.e., NDVI), and frost (i.e., temperatures below 0°C for at least one hour) (Table S1). As a measure of heat accumulation, GDD measures the length of the growing season, which determines the amount of time there are favorable conditions for growth and development in plants and insects (Chuine, 2010; Régnière et al., 2012). Growing degree‐days (GDD) were modeled using a 10°C base (see Table S1 for calculation), which is a commonly used threshold among butterflies and other insects (Cayton et al., 2015; Nufio et al., 2010). Other variables are related to primary production and resource availability such as NDVI and precipitation. The impact of frost events was assessed since they were previously hypothesized to be a contributing factor for the range expansion of *P. cresphontes* (Finkbeiner et al., 2011). Specifically, we tested different frequencies of frost events: the number of frost‐free days and duration of the frost‐free period (Table S1). The effect of the intensity of frost events (i.e., how cold it was during the frost event) could not be tested since the necessary climatic variables were not available. All climatic data were downloaded at a 1‐km resolution and restricted to 1980–2010. All data were downloaded at a North American extent, with the exception of Normalized Difference Vegetation Index (NDVI), which was global in extent.

For the mechanistic models, we created variables for both range extents based on the results from the cold tolerance experiments and minimum daily temperature data from Daymet (Table S1). Three variables were built for each extent by summing the number of days in September and October with mean daily temperatures below the following thresholds: 2.14°C (i.e., CT_min_), −6.6°C (i.e., the SCP from the August generation), and −8°C (i.e., potential lower lethal limit) for each year of the study period 1980–2010 (Table S1). We did not include August in the derivation of these variables because there were 0 days below 2.14°C (CT_min_) between 1980 and 2010 (YOW airport weather station) at the northern range limit (Ottawa, ON). We note that we also derived these variables using minimum daily temperatures for the same time period and results were identical (Table S4). The SCP from the August generation was chosen since it is the one most likely to match when larvae are more likely to experience low temperatures.

Variables were created for both range extents and modeling approaches and then screened for collinearity (Table S5). To reduce collinearity among variables, a variance inflation factor (VIF) analysis from the car package (Fox et al., 2007) with a threshold of 10 was conducted. This threshold was chosen instead of a more restrictive one (e.g., 3 or 5) as it provides a compromise between collinearity and model usefulness (O'brien, 2007). The test was repeated for each of the model extents and approaches (*n* = 4). With the VIF test, the initial list of 16 variables was reduced to eight for the northern range and six for the full range model (Table S5). The final list of variables used for the correlative modeling approach was extreme maximum temperature, precipitation as snow, precipitation, GDD, NDVI, and mean temperature of the coldest month (Table S5). Additionally, for the mechanistic models, CT_min_ and the potential lower lethal limit temperature were also included.

#### 2.3.3 Modeling

Models were built using Maxent (version 3.4.1, Phillipset al., 2006) with the BIOMOD2 package (Thuiller et al., 2016) in R (version 3.41.1). Maxent is a commonly used species distribution modeling technique for presence‐only data (Merow et al., 2013). Maxent uses a maximum‐entropy approach to model species distributions. It relates the species’ presences and pseudo‐absences to the environmental variables to predict where the habitat is suitable across the mapped geographical space. Maxent is one of the more common species distribution modeling software (Elith et al., 2011), and it performs very well compared with other modeling techniques when dealing with presence‐only data (Hernandez et al., 2006).

To model the range, we included hinge features, which allow nonlinear relationships, in the model and allowed the model the possibility of clamping (i.e., the default). Clamping serves to limit how much leniency there is in the range of values projected compared with what was observed. This action is important when predicting into novel geographic space (Stohlgren et al., 2011). However, since the training data and the projection extent were the same, no clamping was done. For each model extent and approach, a background extent was created using the minimum convex polygon function in R (Package adehabitatHR v0.4.16; Calenge, 2006). Pseudo‐absences were randomly generated within the minimum convex polygons (*n* = 10,000; default value).

The models were calibrated using fivefold cross‐validation (i.e., the observations were divided into five (*k* = 5) groups, or folds, of equal size). In cross‐validation, the first fold is treated as a validation set, and the model is fit on the remaining *k* − 1 folds (James et al., 2013). Iterations were limited to 5,000 to leave enough time for model convergence. All other parameters were left at default values.

Models were evaluated using three metrics that assess various aspects of accuracy and discrimination (i.e., the ability of the model to distinguish between suitable and nonsuitable habitat): area under the curve (AUC) of the receiver operating characteristics, kappa, and true skill statistic (TSS). AUC characterizes the model's ability to correctly predict if a presence or absence is a true presence or absence. A value below 0.5 means that the model is no better at predicting occurrences than random, whereas a value of 1 would mean the model predicts all presences/absences perfectly (Yackulic et al., 2013). Although it is the most commonly used metric for assessing model accuracy of species distribution models (Yackulic et al., 2013), it has been criticized for being unreliable when sample sizes are small and due to its reliance on the number of background points. As such, in these contexts, AUC should mainly be used to compare models built with the same variables and occurrences.

The discrimination of the models was further tested using kappa and TSS. These two metrics use confusion matrices to compare models' abilities to predict occurrences correctly (Allouche et al., 2006). While kappa has been shown to be more sensitive to prevalence (i.e., the proportion of locations that are occupied), TSS provides an alternate validation metric that is independent of prevalence (Allouche et al., 2006, but see Somodi et al., 2017). Kappa scores between 0.4 and 0.6 indicate fair agreement, 0.6 and 0.8 indicate moderate agreement, and values greater than 0.8 indicate strong agreement. A TSS score between 0.40 and 0.75 indicates good predictive performance of the model, while a TSS score above 0.75 indicates excellent performance (Allouche et al., 2006).

Models were run 100 times, and the sensitivity (i.e., the proportion of correctly predicted presences) and specificity (i.e., the proportion of correctly predicted absences) were extracted from each iteration and used to calculate mean and standard error of AUC, kappa, and TSS (Cerasoli et al., 2017). The proportion of variation explained by the variables was also extracted from each iteration and averaged across all runs.

#### 2.3.4 Statistical analysis

To determine the role of low temperature in limiting the distribution of *P*. *cresphontes*, model accuracy was compared across the two extents and approaches using *t* tests. Comparisons were based on AUC, kappa, and TSS.

## 3 RESULTS

### 3.1 Cold tolerance experiments

#### 3.1.1 Experiment 1: Supercooling point (SCP) test

The results from the July and August SCP experiments provide support for a chill susceptible strategy in *P*. *cresphontes*. The mean SCP for July was −5.8°C (0.21SE; *n* = 14). There was no significant difference in survival for July larvae that froze compared with those that did not freeze during the SCP experiment (above (i.e., froze): 77% (10/13) vs. below (i.e., did not freeze): 43% (6/14 larvae); *χ* ^2^ = 1.2, *df* = 1, *p*‐value = 0.2). For reasons not known, body mass was not related to SCP (QUBS population: *n* = 13, *F* = 0.43, *p* = .52).

The mean SCP for all August larvae was −6.6°C (0.2SE; *n* = 28), and for the upper 50% of larvae (directly comparable to the July experiment), the mean SCP was −5.9°C (0.17*SE*; *n* = 14). Body mass was related to SCP in the August generation (QUBS population: *n* = 14, *F* = 10.90, *p* = .0063). The August larvae had a 3.5% (1/29) survival rate following freezing (i.e., exposure to temperatures below the mean SCP). The low survival below SCP and apparent lack of change in the SCP suggest that larvae are tolerant of moderate chilling, but intolerant of freezing, and are not undergoing a change in SCP under simulated autumn conditions.

#### 3.1.2 Experiment 2: Low‐temperature survival assays

##### Survival

Our low‐temperature survival assays further demonstrate that larvae are chill‐tolerant or modestly freeze‐avoidant, as they are unable to survive exposure to temperatures below their SCP. In the −2°C test, July larvae had a 100% survival rate (22/22) after 24 hr (Table 1). Therefore, single ecologically relevant exposures to temperatures above their SCP do not have a large impact on larval survival. Moreover, while the survival rate of larvae in July was more affected by exposure to −6°C than −2°C (Table 1), it was still rather high (70%). Therefore, larvae are able to survive a 7‐hr cold exposure near their SCP and thus may be chill‐tolerant or freeze‐avoidant.

**TABLE 1:** Survival rates and developmental success for the low‐temperature assays

Test	Generation	Number of larvae	Larval survival (%)	Pupation (%)	Eclosion (%)
−2°C	July	22^a^	22 (100)	14 (64)	12 (55)
−6°C	July	10	7 (70)	6 (60)	3 (30)
	August	17	17 (100)	17 (100)	0 (0)
−8°C	July	8	1 (12.5)	1 (12.5)	1 (12.5)
	August	10	1 (10)	1 (10)	0 (0)

Note

Test temperature and generation are shown. The percentage of initial larvae that survived the first 24 hr after the experiment, successfully pupated, and eclosed as adults is also shown. All percentages are based on the number of larva that began the experiment.

^a^ This number differs from Table S2 as a larva escaped during the experiment.

August larval survival (100%, 17/17) was significantly higher than that of the July larvae (70%, 7/10) following exposure to −6°C (*χ* ^2^ = 5.1, *df* = 1, *p*‐value = .024; Table 1). This finding provides further evidence in support of a modestly freeze‐avoidant strategy since the mean SCP for the August generation (−6.6°C) was lower than the test temperature (−6°C) and survival was still quite high.

In comparison, in the extreme low‐temperature test (−8°C), larvae had marginal survival in both generations (July: 12.5% (1/8); August: 10% (1/10)) and there was no improvement in survival across generations (Fisher's odds ratio = 0.81, *p*‐value = .99; Table 1). This low survival further suggests larvae may use a freeze‐avoidant strategy since this temperature is below the mean SCP measured in the August generation.

##### Developmental success

Overall, low‐temperature exposure impacted pupation and adult eclosion. The percentage of larvae that successfully pupated and eclosed generally decreased as the temperatures got lower (Table 1). The percentage of larvae that successfully pupated at −8°C was low (July: 12.5% (1/8); August: 10% (1/10)). None of the August pupae that overwintered emerged in the spring. However, since these larvae were exposed to natural ambient conditions and the experimental low temperatures before pupation, it is impossible to determine exact causes of death (e.g., severe sublethal effect of cooling). Nevertheless, although larval survival can be high after exposure to low temperatures, chilling also had effect on developmental success, which is indicative of sublethal chilling injury occurring, even in the absence of freezing.

#### 3.1.3 Experiment 3: CT_min_

The average CT_min_ of larvae was 2.14°C (0.26 *SE*; *n* = 20).

### 3.2 Distribution modeling

#### 3.2.1 Model performance

**FIGURE 3:**
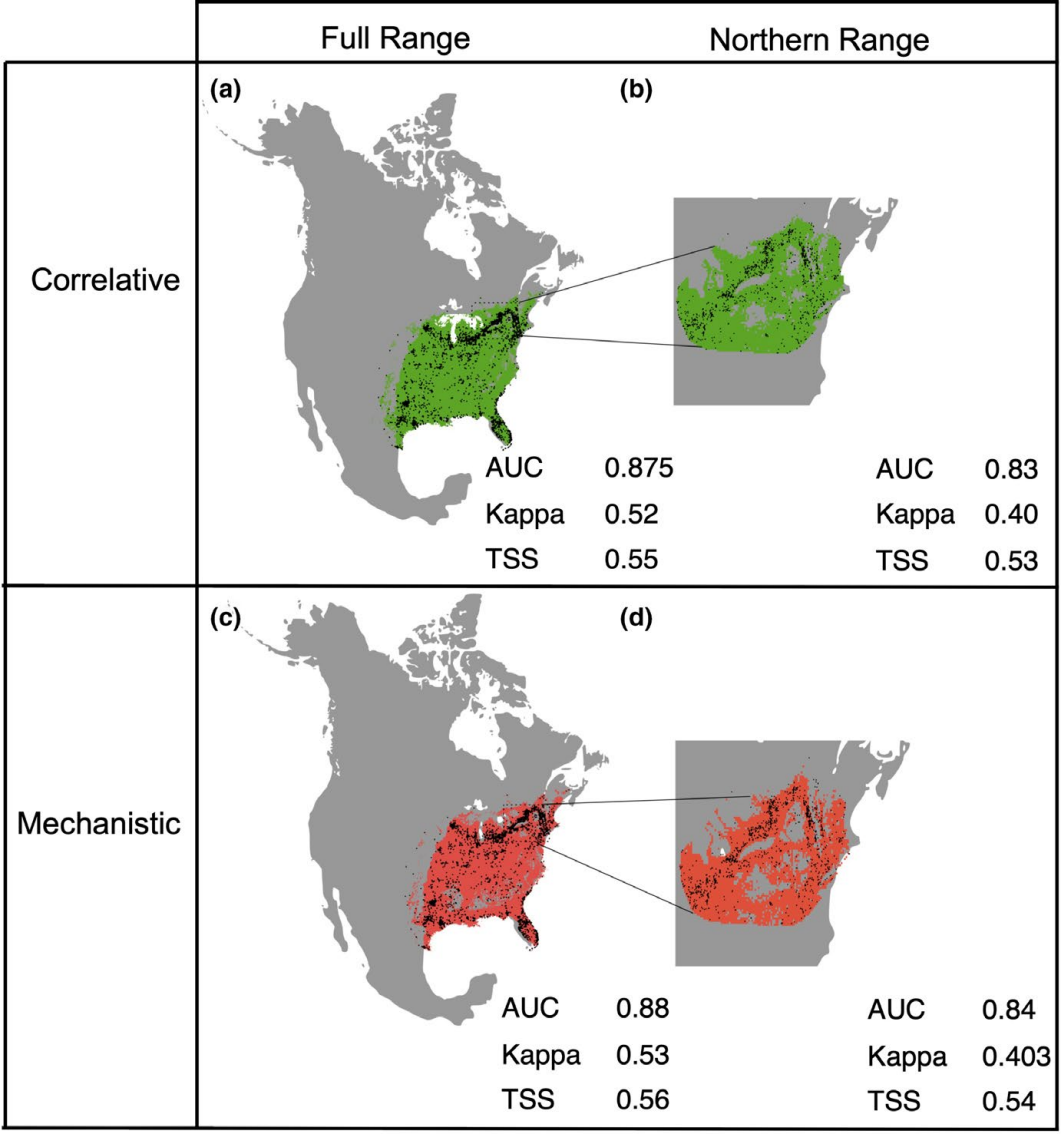
A comparison of spatial predictions and model accuracy for the two model extents (full and northern range) and approaches (correlative and mechanistic) based on the evaluation metrics (AUC (area under the receiver operating characteristic curve), kappa, TSS (true skills test)). Mean accuracy scores across 100 iterations are shown. Statistical comparisons can be found in Tables S6 and S7

Model predictive ability varied depending on the metric of accuracy used. All models were considered good based on TSS (i.e., between 0.4 and 0.75) and AUC (>0.8), whereas based on kappa, the northern range models were weak (<0.4) and the full range models were fair (>0.5; Figure 3).

Statistically, the mechanistic models were significantly more accurate than the correlative models in most but not all cases (Table S6; Figure 3). However, in the cases where there was a significant difference between modeling approaches, the effect size was small (difference in score ranged from 0.003 to 0.008; Table S6), implying the difference is unlikely to be biologically significant. Moreover, false negatives occurred with both modeling approaches (Figure 3). Therefore, it is unlikely the inclusion of cold tolerance metrics significantly improves the prediction of habitat suitability of *P*. *cresphontes*.

The accuracy of the full range models was significantly higher than the accuracy of the northern range models for all evaluation metrics and approaches (Figure 3; Table S7). Although the effect sizes were also not large, they were greater (difference in score ranged from 0.02 to 0.12) and the significant difference in accuracy was consistent across all comparisons (Table S7; Figure 3). Therefore, the habitat suitability of *P*. *cresphontes* is predicted more accurately across its full range than its northern range.

#### 3.2.2 Variable importance

Growing degree‐days was the most important factor explaining the habitat suitability of *P*. *cresphontes* across all model extents and approaches, explaining between 27% and 35% of the variation in habitat suitability (Table 2). Precipitation was the second most important factor across both extents and approaches. Across the full and northern range extents, GDD and precipitation combined explained the majority of the variation in habitat suitability (~50%; Table 2). The physiologically derived variables included in the final model (i.e., CT_min_ and the potential lower lethal limit) only explained a small part of variation in habitat suitability (~7.5%) and ranked 5th and 6th in model contribution (Table 2).

**TABLE 2:** Contribution of the environmental variables in explaining habitat suitability across different model extents and approaches

Variables	North America				Northern range			
	Correlative		Mechanistic		Correlative		Mechanistic	
	Rank order	Variation explained (*SE*)	Rank order	Variation explained (*SE*)	Rank order	Variation explained (*SE*)	Rank order	Variation explained (*SE*)
**Growing degree‐days**	**1**	**31.33 (0.19)**	**1**	**27.34 (0.23)**	**1**	**37.17 (0.08)**	**1**	**35.03 (0.06)**
Precipitation	2	24.87 (0.08)	2	21.70 (0.085)	2	23.94 (0.08)	2	22.47 (0.07)
Extreme maximum temperature	3	17.54 (0.06)	3	16.22 (0.06)	6	2.38 (0.02)	7	2.41 (0.02)
Normalized Difference Vegetation Index	4	15.45 (0.10)	4	15.87 (0.11)	4	16.77 (0.11)	4	14.89 (0.11)
Mean temperature of the coldest month	NA	NA	NA	NA	3	16.9 (0.06)	3	15.57 (0.05)
Precipitation as snow	5	10.80 (0.07)	5	10.05 (0.07)	5	2.84 (0.02)	6	3.46 (0.02)
CT_min_ ^a^	NA	NA	NA	NA	NA	NA	5	5.97 (0.03)
Potential lower lethal temperature^a^	NA	NA	6	8.80 (0.04)	NA	NA	8	0.16 (0.004)

Note

Shown is the rank order of variable importance and mean (±*SE*) proportion of variance explained. In bold is the variable that explains the most amount of variation for each model type.

^a^ Variable derived experimentally.

## 4 DISCUSSION

Although insects are likely to be particularly vulnerable to low temperatures during autumn, no study, to our knowledge, has considered low temperatures during autumn as a limiting factor on the geographic distributions of insects. Here, we test this hypothesis at the northern edge of the distribution of the widespread butterfly, the Giant Swallowtail, *P*. *cresphontes*. Our study contributes three main findings that lead to a rejection of this hypothesis. First, given the survival of larvae to prolonged exposures to temperatures close to but not below their SCP, they are chill‐tolerant or modestly freeze‐avoidant at their northern range limit at this time of year. Their low survival below the SCP provides support that this species is unable to handle freezing before pupation and that they may use a freeze‐avoidant strategy. However, further testing for seasonal plasticity in the SCP is required to confirm a freeze‐avoidant strategy as we had limited power and were unable to directly compare the change in SCP between the two generations. An active depression of the SCP over the season would indicate that larvae are accumulating cryoprotectants and that the larval cold tolerance could be increasing, thus supporting a freeze‐avoidant strategy.

Our results demonstrate that ecologically relevant exposures to temperatures above the SCP are not lethal, but below the SCP are, thus providing a clear picture of the lower end of thermal tolerance for this species. This means that for *P*. *cresphontes* larvae, a single overnight exposure to temperatures near −8°C will impede their survival and could represent a potential lower lethal limit. Consequently, areas with early autumn temperatures that reach −8°C should be inhospitable for *P*. *cresphontes*. However, in the Ottawa region, which is at the northern range limit, there are only 0–3 frost episodes on average from September to October. Therefore, low temperatures (i.e., <−7°C) during early autumn are unlikely to be experienced by the larvae there or anywhere in its current range. This suggests that a single exposure to low temperatures in early autumn is unlikely to limit the current northern range of *P*. *cresphontes* through cold‐induced mortality. However, there may be sublethal effects of chilling (e.g., effects on developmental success), which could still impact overall insect fitness.

Second, we found that exposure to normal frost temperatures for this time of year in this area (i.e., −1°C to −2°C), which are above the SCP, did not affect larval survival. Therefore, it is also unlikely that frost in early autumn is a limiting factor of *P*. *cresphontes* northern range. This is in concordance with the field observations from Finkbeiner et al. (2011), which showed that *P*. *cresphontes* larvae can survive frost events. Since larvae had no problem reaching pupation after exposure to −2°C, the most common frost temperature, and the majority of larvae still successfully pupated (60%) after the low frost temperature test (i.e., −6°C), the cold tolerance of larvae at the current northern range limit seems sufficient to cope with the climate they experience. However, further testing in ambient conditions and with a proper control is needed to determine whether cold exposure on larvae have significant sublethal effects on eclosion as rates of eclosion in this experiment were less than 30%, but a control group was not included due to sample size constraints. It has been demonstrated elsewhere that the success of each life stage matters in the response of butterfly species to climate change (Radchuk et al., 2013).

Third, the species distribution modeling results were consistent with the experimental results: Cold‐related variables did not explain the distribution of *P*. *cresphontes* at a broad scale. Together, these results provide strong support that exposure to temperatures above −6.6°C during autumn does not limit the northern range of *P*. *cresphontes*. Instead, growing degree‐days and precipitation are the most important predictors, of those tested here, on the distribution of *P*. *cresphontes* at a broad scale. While other factors were not tested here, for example, biotic interactions (e.g., host plant occurrence; Filazzola et al., 2020), our results suggest that *P*. *cresphontes* depends on specific heat accumulation and water availability to complete its life cycle. These factors have also been identified as important in predicting the range of other butterflies (Eskildsen et al., 2013; Luoto et al., 2006). Evidence suggests there is a strong relationship between the number of growing degree‐days and the growth rate of larvae, and the foraging activities of adults (Kukal & Dawson, 1989; Ritland & Scriber, 1985; Schneider & Root, 2002). Our result is consistent with studies showing the constraints of growing degree‐days on size and voltinism for species with a longer larval development time in relation to growing season length (Blanckenhorn & Demont, 2004; Horne et al., 2015; Kivelä et al., 2016; Roff, 1980). Precipitation can limit the range of insects directly due to dehydration or indirectly by imposing limitations on primary productivity, which in return transposes to higher resource availability.

The full range model was more accurate at predicting the distribution of *P*. *cresphontes* than the northern range model. Similar results have been found for other species, with model accuracy and performance increasing with larger study extents (Connor et al., 2019; VanDerWal et al., 2009). This is likely a result of smaller environmental gradients in the northern range, which causes the model to have a greater difficulty detecting differences between presences and absences, and in turn leads to difficulties predicting presences and absences in novel areas (Smith & Santos, 2019). Prevalence can also decrease with larger study extents (Barbet‐Massin et al., 2012). However, the accuracy of the two models was still different using TSS, a metric that is not as sensitive to prevalence (Table S6; Figure 3; Somodi et al., 2017). Nevertheless, even if model performance was lower in the northern range relative to the full range, the model was still valid and performed adequately across all metrics.

While growing degree‐days and precipitation explain a lot of the variation in habitat suitability for *P*. *cresphontes*, we may have underestimated the role of cold tolerance at the northern range edge for four reasons. First, low‐temperature thresholds, such as the CT_min_, although not lethal, could still impact *P*. *cresphontes* survival in natural conditions due to the enhanced risk of predation or starvation linked with the loss in mobility at temperatures below 2°C. While a possibility, this hypothesis depends on a high likelihood of predation, which is unknown across *P*. *cresphontes* range (Hazel et al., 1998; McAuslane, 2009). Low temperature may also lead to sublethal effects on developmental success, energy status, and behavior. Second, it is also possible that more prolonged or repeated exposure to low temperatures could impact *P*. *cresphontes* larval survival or have sublethal effects at the northern range edge because we did not test for the effects of repeated cold injury or chilling. In *Drosophila*, multiple cold events could cause an accumulation of cold injuries resulting in larval death or in sublethal effects such as decreased feeding, fecundity, and dispersal ability (Marshall & Sinclair, 2012).

Third, the SCP estimated here is effectively a theoretical one. Since we could not collect and test the last emerging larvae of the season due to the difficulty in finding them, they might not have reached their peak cold tolerance, and therefore, in situ SCP could be lower (i.e., their cold hardiness may have been underestimated). Moreover, as we did not test the cold tolerance of the pupae, the life stage most likely to experience low temperatures in this region, their SCP could be lower. On the other hand, the in situ SCP may be higher since environmental factors in natural conditions, such as air moisture or exposure to ice nucleators, may further affect survival at temperatures above SCP. Nevertheless, because the low‐temperature thresholds derived experimentally in this study could be an underestimate, the mechanistic models may not have been optimized perfectly, thus underestimating the role of cold tolerance metrics at a broad scale. Fourth, the low sample size in our experiments may have limited our power; however, the overall conclusions of the study are unlikely to be affected. Since temperatures below the SCP (i.e., <−7°C) are very unlikely to occur multiple times before pupation, if at all, low temperatures during autumn remain unlikely to limit the northern range of *P*. *cresphontes*.

Given our lack of knowledge about the overwintering ecology and physiology of this species in its northern range, pupal overwinter survival may be a more important limiting factor for the species’ northern range limit. Unfortunately, it is unknown whether they overwinter in the leaf litter on the ground or tree branches above the ground. If on the ground, they would likely be covered in snow (average annual snowfall in Ottawa is 224 cm [Environment and Climate Change Canada]) and therefore buffered against low temperatures. The temperature 30 cm below snow cover is usually −4°C (Flin & Brzoska, 2008). This behavior would increase their chances of surviving the winter. Indeed, West and Hazel (1996) showed that in Virginia, *P*. *cresphontes* pupate 3–25 cm off the ground on dead branches. In the northern range, it is likely that even at these heights, chrysalids would be covered by snow. While ontogenetic variation in thermal tolerance is present in other species (Terblanche et al., 2006; Marais et al., 2009; MacLean et al., 2016; but see Ouimette, 2018) and only larvae were tested in this study, *P*. *cresphontes* larvae purge their food and liquids before pupation; thus, they are likely to be even more cold‐tolerant at the pupal stage. Other members of *Papilio* are more cold‐tolerant than what we determined for the larval stage of *P*. *cresphontes*. *P*. *canadensis* and *P*. *glaucus* are considered to be freeze‐tolerant at the pupal stage and have an SCP of −23°C to −27°C (Kukal et al., 1991). *P*. *xuthus* is freeze‐avoidant with an SCP of −25°C (Shimada, 1988). Therefore, the pupae of *P*. *cresphontes* are likely to have even greater cold hardiness than the larvae and thus be able to survive the winter at the northern range if they pupate at similar heights as the populations in Virginia. Nevertheless, future studies should determine which temperature conditions pupae experience in the winter and use these temperatures to guide the design of experiments to determine cold tolerance plasticity.

Finally, our study has implications for climate change‐driven range expansion in the species. First, our results suggest that the warming of autumn temperatures over the past decade is unlikely to have been the factor that led to the recent range expansion of *P*. *cresphones* into Eastern Ontario, at least via low‐temperature limits, as originally hypothesized by Finkbeiner et al. (2011). Instead, the warming of winter temperatures could have ameliorated overwintering survival, leading to an improvement in habitat suitability. Alternatively, warming autumn temperatures could have increased growing season length, increasing the time available for resource acquisition and development (Blanckenhorn & Demont, 2004), and increasing voltinism, as has been observed in other butterfly species in North America (Zografou et al., 2021). However, based on expert knowledge (i.e., Toronto Entomologists’ Association), *P*. *cresphontes* has been bivoltine since it first expanded into the Ottawa region so a change in voltinism is unlikely to have been the key factor that led to recent range expansion. Future northward range expansion beyond the Ottawa region could be constrained by the distribution, and response, of its main host plant in the area (*Z*. *americanum*) to climate change, which is unknown. Based on regional floras (Rousseau, 1974; Soper & Heimburger, 1990), the northern range limit of *Z*. *americanum* is thought to occur just north of Ottawa; however, recent vegetation surveys beyond the region have not been done.

## 5 CONCLUSIONS

Our results demonstrate that the cold tolerance of *P*. *cresphontes* larvae is well matched to its environment in the northern part of its range and that it is unlikely that low temperatures in autumn are limiting its range. Further study on determining the key factor(s) limiting the northern range of *P*. *cresphontes* should focus on defining the cold hardiness of the pupal stage, the overwintering behavior, and the ecological relevance of the physiological thresholds found in this study. Determining the ultimate factors that limit species’ distributions will be critical in accurately predicting species’ range shifts in response to future climate change.

## CONFLICT OF INTEREST

None declared.

## AUTHOR CONTRIBUTIONS

**Philippe Tremblay:** Conceptualization (supporting); Data curation (lead); Formal analysis (lead); Investigation (supporting); Methodology (supporting); Visualization (equal); Writing‐original draft (lead); Writing‐review & editing (supporting). **Heath A**. **MacMillan:** Conceptualization (supporting); Methodology (supporting); Visualization (supporting); Writing‐review & editing (supporting). **Heather M. Kharouba:** Conceptualization (lead); Formal analysis (supporting); Funding acquisition (lead); Investigation (lead); Methodology (equal); Project administration (lead); Supervision (lead); Visualization (equal); Writing‐original draft (supporting); Writing‐review & editing (lead).

## Supporting information

Appendix S1

## Data Availability

Data supporting the results are archived in Dryad, accessible at https://doi.org/10.5061/dryad.cfxpnvx52.
