# Supplementary material for: Autumn larval cold tolerance does not predict the northern range limit of a widespread butterfly species
Source: Ecol Evol. 2021 May 22;11(12):8332–46. doi: 10.1002/ece3.7663 (PMC8216912; doi:10.1002/ece3.7663)
Supplement: Supplementary file 1 — Appendix S1 [file ECE3-11-8332-s001.docx]

**Appendix S1.** Additional results.

1. Table S1. List of alternative hypotheses about factors liming the distribution of *P. cresphontes*
2. Table S2. Number of larvae among generations and sites for the experiments.
3. Table S3. A comparison of larval survival rate and the rates of pupation and adult emergence across sites for the low-temperature assays.
4. Table S4. Results of a sensitivity analysis where mechanistic variables were built using minimum daily temperatures below the cold tolerance thresholds.
5. Table S5. Results from the variance inflation factor (VIF) analysis used in the species distribution modelling.
6. Table S6. Comparison of the accuracy of species distribution models between modelling approaches.
7. Table S7. Comparison of the accuracy of species distribution models between spatial extents.
8. Figure S1. Experimental details for cold tolerance tests.

Table S1. List of alternative hypotheses about factors liming the distribution of *P. cresphontes*. The data source is shown as well as the calculation used to produce the final rasters. All variables are averaged over the period from 1980-2010 and the original map projection of all variables was Lambert Conical Conic except for NDVI, which was sinusoidal.

| **Acronym** | **Full name (units)** | **Data source** | **Calculation** | **Importance** |
| --- | --- | --- | --- | --- |
| NDVI | Normalized Difference Vegetation Index | Modis/Terra project | Averaged monthly NDVI raster’s over the time frame. | Seto 2004, Pettorelli 2005 |
| bFFP | beginning of frost free period (FFP; day of year) | Databasin | The day of the year on which FFP begins | Hayes 1982, Westwood & Blair 2010 |
| eFFP | end of frost free period (day of year) | Databasin | The day of the year on which FFP ends | Hayes 1982, Westwood & Blair 2010 |
| FFP | frost free period (days) | Databasin | The number of days between the last spring frost and the first autumn frost | Hayes 1982, Westwood & Blair 2010 |
| NFFD | number of frost free days | Databasin | Number of days above 0°C. | Hayes 1982, Westwood & Blair 2010 |
| PAS | average precipitation as snow (mm) | Databasin | Accumulated snowfall averaged across the time frame | Roland & Matter 2016 |
| Precip | average precipitation as rainfall (mm) | Databasin | Accumulated rainfall averaged across the time frame | Storch et al. 2003 |
| EMT | extreme minimum temperature (°C) | Databasin | Lowest temperature recorded for every given year, averaged over the timeframe. | Crozier 2003 |
| EXT | extreme maximum temperature (°C) | Databasin | Highest temperature recorded for every given year, averaged over the timeframe. | Malcolm et al. 1987 |
| MCMT | mean temperature of the coldest month (°C) | Databasin | Average daily temperature for the coldest month | Crozier 2003; Luoto et al., 2006 |
| MWMT | mean temperature of the warmest month (°C) | Databasin | Average daily temperature for the warmest month | Crozier 2003 |
| octtre | average temperature in October (°C) | Daymet | Average daily temperature for October. | Larsen & Lee 1994 |
| GDD | average growing degree-days of base 10°C | Daymet | ((Tmax + Tmin) / 2) -10 | Luoto et al., 2006 |
| SCP | average number of days per year below -6.6°C (days) | Daymet | Based on the experimentally derived SCP; a day was counted if the average daily tem.perature reached -6.6°C. Days were counted and averaged across time frame. Only September and October considered. | Ungerer et al., 1999 |
| CT_min_ | average number of days per year below 2.14°C (days) | Daymet | Based on the experimentally derived CT_min_; a day was counted if the average daily temperature reached 2.14°C. Days were counted and averaged across time frame. Only September and October considered. | Andersen et al. 2015 |
| PLLT | Potential lower lethal temperature; Average number of days per year below -8°C (days) | Daymet | Based on the experimentally derived potential lower lethal temperature; a day was counted if the average daily temperature reached -8°C. Days were counted and averaged across time frame. Only September and October considered. | Andersen et al. 2015 |

Table S2. Number of larvae among generations and sites for the three experiments. The sites are: Queen’s University Biological Station (QUBS), Mud lake, Shirley’s Bay and Brockville.

| Generation | Experiment | Temperature treatment (°C) | Site | Number of larvae |
| --- | --- | --- | --- | --- |
| July | SCP | | QUBS | 27 |
|  | Low temperature | -2 | QUBS | 15 |
|  |  |  | Shirley’s Bay | 2 |
|  |  |  | Brockville | 6 |
|  |  | -6 | Mud lake | 10 |
|  |  | -8 | Mud lake | 8 |
|  | CT_min_ | | QUBS | 20 |
| August | SCP (total n=29) | | QUBS | 14 |
|  |  |  | Mud lake | 5 |
|  |  |  | Brockville | 3 |
|  |  |  | Shirley’s Bay | 7 |
|  | Low temperature | -6 | QUBS | 8 |
|  |  |  | Mud lake | 5 |
|  |  |  | Shirley’s Bay | 4 |
|  |  | -8 | QUBS | 2 |
|  |  |  | Mud lake | 5 |
|  |  |  | Shirley’s Bay | 3 |

Table S3. A comparison of larval survival rate and the rates of pupation and adult eclosion across sites for the low-temperature assays (i.e., -2°C, -6°C, -8°C tests). The results from χ2 goodness-of-fit tests are shown. The NAs are in cases where all individuals for a given test were from the same site or the test was not repeated for both generations. See Table S1 for the number of larvae across sites.

| Test | Generation | Life stage | χ2 | Degrees of freedom | p value |
| --- | --- | --- | --- | --- | --- |
| -2°C | July | Larval | 0.40 | 2 | 0.40 |
|  |  | Pupal | 2.41 | 2 | 0.30 |
|  |  | Adult | 0.93 | 2 | 0.63 |
|  | August | Larval | NA | NA | NA |
|  |  | Pupal | NA | NA | NA |
|  |  | Adult | NA | NA | NA |
| -6°C | July | Larval | NA | NA | NA |
|  |  | Pupal | NA | NA | NA |
|  |  | Adult | NA | NA | NA |
|  | August | Larval | 1.53 | 2 | 0.47 |
|  |  | Pupal | NA | NA | NA |
|  |  | Adult | NA | NA | NA |
| -8°C | July | Larval | NA | NA | NA |
|  |  | Pupal | NA | NA | NA |
|  |  | Adult | NA | NA | NA |
|  | August | Larval | 4.44 | 2 | 0.11 |
|  |  | Pupal | NA | NA | NA |
|  |  | Adult | NA | NA | NA |

Table S4. Results of a sensitivity analysis where mechanistic variables were built by summing the number of days for September and October months using minimum daily temperatures below the cold tolerance thresholds. Contribution of the environmental variables in explaining habitat suitability across different model extents and approaches. Shown is the rank order of variable importance and mean (± S.E) proportion of variance explained. In bold is the variable that explains the most amount of variation for each model type.

| Variables | North America | | | | Northern range | | | |
| --- | --- | --- | --- | --- | --- | --- | --- | --- |
|  | Correlative | | Mechanistic | | Correlative | | Mechanistic | |
|  | Rank order | Variation explained (SE) | Rank order | Variation explained (SE) | Rank order | Variation explained (SE) | Rank order | Variation explained (SE) |
| **Growing degree days** | **1** | **31.33 (0.19)** | **1** | **27.02 (0.23)** | **1** | **37.17 (0.08)** | **1** | **35.02 (0.06)** |
| Precipitation | 2 | 24.87 (0.08) | 2 | 23.16 (0.085) | 2 | 23.94 (0.08) | 2 | 22.43 (0.07) |
| Extreme maximum temperature | 3 | 17.54 (0.06) | 3 | 16.09 (0.06) | 6 | 2.38 (0.02) | 7 | 2.40 (0.02) |
| Normalized Difference Vegetation Index | 4 | 15.45 (0.10) | 4 | 15.23 (0.11) | 4 | 16.77 (0.11) | 4 | 14.94 (0.11) |
| Mean temperature of the coldest month | NA | NA | NA | NA | 3 | 16.9 (0.06) | 3 | 15.55 (0.05) |
| Precipitation as snow | 5 | 10.80 (0.07) | 5 | 10.19 (0.07) | 5 | 2.84 (0.02) | 6 | 3.49 (0.02) |
| Chill coma* | NA | NA | NA | NA | NA | NA | 5 | 5.98 (0.03) |
| Potential lower lethal temperature* | NA | NA | 6 | 8.60 (0.04) | NA | NA | 8 | 0.16 (0.004) |

*mechanistic variable; derived experimentally

Table S5. Results from the variance inflation factor (VIF) analysis conducted on the 16 climatic variables used in the species distribution modelling for the two different extents. Shown here are the variables included in the final models based on a threshold of 10 (i.e., those with a score below 10). Variables that were ‘excluded’ had a VIF above 10.

| Extent | Variables | VIF values |
| --- | --- | --- |
| Northern range | Normalized Difference Vegetation Index | 2.03 |
|  | Extreme maximum temperature (°C) | 3.08 |
|  | Precipitation as snow (mm) | 1.16 |
|  | Growing degree-days | 5.55 |
|  | Potential lower lethal temperature (days)* | 1.53 |
|  | Mean temperature of the coldest month (°C) | 5.59 |
|  | Precipitation (mm) | 1.77 |
|  | CT_min_ (days)^§^ | 6.95 |
| Full range | Normalized Difference Vegetation Index | 2.48 |
|  | Extreme maximum temperature (°C) | 3.59 |
|  | Precipitation as snow (mm) | 2.80 |
|  | Growing degree-days | 2.02 |
|  | Potential lower lethal temperature (days)* | 1.21 |
|  | Precipitation (mm) | 3.31 |

*Average number of days per year below -8°C

^§^Average number of days per year below 2.14.

Table S6: Comparison of the mean accuracy of species distribution models with and without mechanistic variables at two spatial extents: northern range and full range. Shown are the t-test results comparing AUC (the area under the receiver operating characteristic curve) and TSS (true skill statistic). The comparisons in bold are statistically significant (p<0.05).

| Extent | Metric | Mean difference in score* | t-value | p-value | Degrees of freedom |
| --- | --- | --- | --- | --- | --- |
| Northern range | **AUC** | **0.0030** | **2.59** | **0.01** | **999.32** |
|  | **Kappa** | **0.0034** | **2.74** | **0.006** | **999.81** |
|  | **TSS** | **0.0078** | **4.75** | **<0.001** | **999.23** |
| Full range | **AUC** | **0.0074** | **2.74** | **0.006** | **999.95** |
|  | **Kappa** | **0.0069** | **2.69** | **0.007** | **999.59** |
|  | TSS | 0.0038 | 1.58 | 0.11 | 999.41 |

* Mean= mechanistic score - correlative sore

Table S7: Comparison of the accuracy of species distribution models between the two extents (northern range and full range) and for both approaches (correlative or mechanistic). Shown are the t-test results comparing AUC (the area under the receiver operating characteristic curve) and TSS (true skill statistic). The comparisons in bold are statistically significant (p<0.05).

| Approach | Metric | Mean difference in score* | t-value | p-value | Degrees of freedom |
| --- | --- | --- | --- | --- | --- |
| Correlative | **AUC** | **0.041** | **8.37** | **<0.001** | **957.11** |
|  | **Kappa** | **0.12** | **72.98** | **<0.001** | **958.51** |
|  | **TSS** | **0.023** | **9.32** | **<0.001** | **957.4** |
| Mechanistic | **AUC** | **0.046** | **24.37** | **<0.001** | **977.72** |
|  | **Kappa** | **0.12** | **63.46** | **<0.001** | **950.57** |
|  | **TSS** | **0.019** | **8.53** | **<0.001** | **958.14** |

* Mean= Full range score – Northern range score

Figure S1. Experimental details for cold tolerance tests. Overview of starting and testing temperatures and dates of collection and tests for July (a) and August (b) generation. Only first collection date and only rough test dates are shown to improve visualization. ‘doy’ refers to the day of year. (c) Profile of temperature conditions in the environmental chamber for larvae from the August generation. Shown is the diurnal temperature range the chamber was programmed for a given week. Parameters were modified weekly to match the conditions from August to October (meteomedia.ca).

References

Andersen, J. L., Manenti, T., Sørensen, J. G., MacMillan, H. A., Loeschcke, V., & Overgaard, J. (2015). How to assess *Drosophila* cold tolerance: chill coma temperature and lower lethal temperature are the best predictors of cold distribution limits. *Functional Ecology*, *29*, 55–65. <https://doi.org/10.1111/1365-2435.12310>

Crozier, L. (2003). Winter warming facilitates range expansion: cold tolerance of the butterfly Atalopedes campestris. *Oecologia*, *135*(4), 648–656. https://doi.org/10.1007/s00442-003-1219-2

Hayes, J.L. (1982). A study of the relationships of diapause phenomena and other life history characters in temperate butterflies. *The American Naturalist*, *120*(2), 160-170.

Larsen, K. J., & Lee, R. E. (1994). Cold tolerance including rapid cold-hardening and inoculative freezing of fall migrant monarch butterflies in Ohio. *Journal of Insect Physiology*, *40*(10), 859–864. https://doi.org/10.1016/0022-1910(94)90019-1

Luoto, M., Heikkinen, R. K., Pöyry, J., & Saarinen, K. (2006). Determinants of the biogeographical distribution of butterflies in boreal regions. *Journal of Biogeography*, *33*(10), 1764–1778. https://doi.org/10.1111/j.1365-2699.2005.01395.x

Malcolm, S.B., Cockrell, B.J. and Brower, L.P. (1987). Monarch butterfly voltinism: effects of temperature constraints at different latitudes. *Oikos*, 77-82. https://doi.org/10.2307/3565556

Pettorelli, N., Vik, J.O., Mysterud, A., Gaillard, J.M., Tucker, C.J. and Stenseth, N.C. (2005). Using the satellite-derived NDVI to assess ecological responses to environmental change. *Trends in ecology & evolution*, *20*(9), 503-510. <https://doi.org/10.1016/j.tree.2005.05.011>

Roland, J., & Matter, S. F. (2016). Pivotal effect of early-winter temperatures and snowfall on population growth of alpine *Parnassius smintheus* butterflies. *Ecological Monographs*, *86*(4), 412–428. https://doi.org/10.1002/ecm.1225

Seto, K.C., Fleishman, E., Fay, J.P. and Betrus, C.J., (2004). Linking spatial patterns of bird and butterfly species richness with Landsat TM derived NDVI. *International Journal of Remote Sensing*, *25*(20), 4309-4324. <https://doi.org/10.1080/0143116042000192358>

Storch, D., Konvicka, M., Benes, J., Martinková, J. and Gaston, K.J., (2003). Distribution patterns in butterflies and birds of the Czech Republic: separating effects of habitat and geographical position. *Journal of Biogeography*, *30*(8), 1195-1205. <https://doi.org/10.1046/j.1365-2699.2003.00917.x>

Ungerer, M. J., Ayres, M. P., & Lombardero, M. J. (1999). Climate and the northern distribution limits of *Dendroctonus frontalis* Zimmermann (Coleoptera: Scolytidae). *Journal of Biogeography*, *26*(6), 1133–1145. <https://doi.org/10.1046/j.1365-2699.1999.00363.x>

Westwood, A.R. & Blair, D. (2010). Effect of regional climate warming on the phenology of butterflies in boreal forests in Manitoba, Canada. *Environmental entomology*, *39*(4), 1122-1133. <https://doi.org/10.1603/EN09143>
